# Leveraging a qualitative data repository to integrate patient and caregiver perspectives into clinical research

**DOI:** 10.1017/cts.2021.822

**Published:** 2021-07-21

**Authors:** Vivian Christensen, Kellee Parker, Erika Cottrell

**Affiliations:** 1 Oregon Clinical and Translational Research Institute (OCTRI), Oregon Health and Science University, Portland, OR, USA; 2 Department of Pediatrics, Division of Pediatric Hematology and Oncology, University of Utah, Salt Lake City, UT, USA; 3 Department of Pediatrics, Division of Pediatric Hematology and Oncology, Oregon Health and Science University; 4 OCHIN, Inc., Portland, OR, USA

**Keywords:** DIPEx, qualitative, patient narratives, pediatric cancer, clinical research, translational research, health experiences

## Abstract

Understanding patient and caregiver experiences is a critical component of the conception, design, and implementation of clinical research studies. The “Database of Individual Patient Experiences” (DIPEx) is an innovative, evidence-based approach for eliciting rich information about health experiences. We conducted a formative evaluation with 14 pediatric oncology researchers to assess the value of using data from a DIPEx study on patient and caregiver experiences with childhood cancer to inform patient-centered research in pediatric oncology. Participants identified barriers to incorporating patient perspectives and experiences into their research and how the DIPEx approach could be leveraged to facilitate this practice.

## Introduction

To align research efforts with the real-world needs of patients and caregivers, there are continued calls to actively engage patients and their caregivers and to incorporate their perspectives, values, and preferences into the conception, design, and implementation of clinical research studies [1–4]. Although recent evidence has demonstrated the value of patient and caregiver engagement, and this practice has continued to increase [5], incorporating patient/caregiver perspectives into clinical research requires sufficient resources and support and is often underutilized [1, 3, 6–8]. For example, a systematic review of research priority activities in pediatric chronic disease, including cancer, found that patient and family engagement in research priority setting was rare, occurring in approximately 25% of studies with only 5% involving input from children directly [8].

In this paper, we explore whether the Database of Individual Patients’ Experiences (DIPEx) – an innovative, evidence-based approach for eliciting rich narratives about health experiences – could be leveraged to facilitate the integration of patient/caregiver perspectives into clinical research. Developed in 2001 by the Oxford University Health Experience Research Group (HERG), the DIPEx model, which is recognized as the “gold standard” for health experiences research in the United Kingdom, utilizes rigorous qualitative methods to collect and analyze interviews about specific health conditions [9, 10]. Interviews about a given health condition are collected until saturation (which is usually reached after ˜35–50 interviews) and every attempt is made to capture the widest range of experiences possible, including experiences of traditionally marginalized underrepresented populations [11]. Since its inception, 15 additional countries have adopted the DIPEx approach. The US chapter, called the Health Experiences Research Network (HERN), was launched in 2014. Originally created as a trusted source of information about patients for patients, caregivers, and clinicians, the DIPEx model includes a commitment to broad dissemination. Findings from interviews about a specific health condition are synthesized through lay language summaries that are illustrated with video, audio, and written clips from in-depth interviews with participants; completed web-based “modules” are presented via a public-facing website. In addition, qualitative data are archived in a repository and available for secondary analysis and use, upon request [10].

DIPEx web-based modules and the associated qualitative data repositories have been deployed for a variety of secondary uses, including the development of medical decision aids [12] and practice guidelines [13, 14], health system redesign initiatives, and modalities for medical education [15–17]. There has been less exploration, however, of the utility of this resource for informing the conception, design, conduct, and dissemination of clinical research [2, 10, 18]. We conducted a formative evaluation among pediatric oncology researchers to understand: (1) their experiences with, and perspectives on, incorporating patient/caregiver perspectives into clinical research and (2) the feasibility and utility of using DIPEx web-based modules and/or raw qualitative data from patient interviews to inform patient-centered research in pediatric oncology.

## Methods

### Data Collection

We conducted semi-structured phone interviews with pediatric oncology researchers. A snowball sampling method was used to recruit researchers from a variety of geographic locations and academic institutions. We initially reached out to seven oncology researchers at Oregon Health & Science University (OHSU), Doernbecher Children’s Hospital (DCH). At the end of each interview, we asked participants to identify others for possible recruitment. Verbal consent was provided prior to the interview, participation was voluntary, and no incentives were provided. Interviews were conducted by members of the research team (VC, KP), audio-recorded, lasted for 20–40 min, and transcribed verbatim. Institutional Review Board approval was obtained from OHSU.

A semi-structured interview guide was developed (Supplement 1: Interview Guide) based on a review of the literature and consultation with researchers at OHSU. Interview questions focused on past experiences using patient/caregiver perspectives in clinical research, perceived barriers and facilitators to engaging patients/caregivers in research, and the utility of leveraging DIPEx web modules and/or data repositories to enhance the patient-centeredness of research. Prior to the interview, participants were provided with a written summary describing DIPEx, and each interview started with a similar overview.

### Data Analysis

All transcripts were independently dual-coded (VC, KP) using inductive thematic analysis [19]. An initial codebook was developed using preliminary themes and subthemes. Codebook refinements were made as new themes and subthemes were identified. Identified themes were deliberated using an iterative process until consensus was reached. Interviews were conducted until no new themes emerged [20].

## Results

Seventeen pediatric oncology researchers were recruited and 14 were ultimately interviewed from 5 different academic institutions (DCH at OHSU (n = 7), Children’s Hospital of Philadelphia (n = 4), Brenner Children’s Hospital at Wake Forest (n = 1), Children’s Hospital of Eastern Ontario at University of Ottawa (n = 1), and Monroe Carell Jr. Children’s Hospital at Vanderbilt n = 1)) between January 24, 2019 and August 9, 2019. Four participants (28%) were male. Participants represented a broad range of clinical research expertise from pediatric oncology fellow to full professor. The following sections broadly describe our findings. See Table 1 for additional exemplar quotes for each identified theme.

**Table 1.** Selected participant quotations

Domain	Identified themes	Representative quotations from participant interviews
Past experiences incorporating patient/caregiver perspectives into clinical research	No experience	• I didn’t know of any resources available to get the information from parents. But I think that it may have been helpful in retrospect (01).
	Some experience	• I have…looked at using patient reported symptom information for clinical management in the inpatient setting…I also have a number of other things looking at the patient experience when undergoing specific types of drug therapies (09).
	Extensive experience	• I participate in research that includes families [as] part of the formula of the process for that research. I’ve met with families to get their perspectives on how the research can be conducted. I’ve met with patient advocates when it is patient-centered research (13).
Barriers to engaging patients/caregivers in research	Lack of expertise on methods for patient/caregiver engagement	• I think the biggest thing is probably education in terms of physicians and clinicians really understanding how to use that information. From my own perspective, I can tell you I don’t have a lot of confidence in my ability to interpret patient data or the qualitative data to really know how to beneficially use it in my research. I think there’s a general lack of knowledge on how to interpret patient opinions and make it something that they can use more broadly (01). • I think an enormous roadblock is the fact that probably patient-centered or patient reports are inherently—well, are extremely valuable but inherently subjective and are difficult data to interpret. And so, I think that those of us who were trained to do research…have much more formal training in quantitative type of research. And I think patient-centered outcomes research tends to lean toward qualitative research, which I’m not at all suggesting one is better than the other. I’m just saying our training and our experience is much more…we’re much more comfortable in the quantitative realm than we are in qualitative research (08).
	Availability of time and resources for patient/engagement in pediatric oncology research	• I think lack of time when you’re doing research is a big impediment to doing anything comprehensive, so if you don’t have a lot of time to sit down and think what you’re designing or you’re on a time crunch to develop a product. I don’t have time to look at all the feedback, I’m just going to go ahead and do it. I think time is a big factor (14). • And so first of all, the time and money related to clinical trials, it is very hard to get funding to do any kind of clinical research. But it’s the rare person who has that expertise to get that external funding…So basically if we only have a certain amount of time in general it’s going to be toward the therapeutics, with better outcomes being the goal. We don’t have the luxury of, in general, doing what we used to be able to do 15–20 years ago which was do all sorts of studies (03).
	Research Priorities	• Frankly, I think it’s never crossed anybody’s mind. I think that as researchers and oncologists, we think we know these diseases so well and we know what’s important, and that’s just "Are patients surviving or not surviving? And isn’t that the most important thing? If a child goes deaf or whatever, what’s the big deal in that? At least, we were able to save your child." And I think that’s sort of the attitude that most people have, that, "Isn’t survival the number one thing that we’re looking out for (05)? • “I think sometimes researchers have their own agenda, not in a bad way, but they have their agenda of where they want a study to go. And, they may be afraid to hear that patients might feel differently about where that study should go. So, maybe there is some concern that if they need to incorporate those perspectives as they develop a study, it could slow down the development or end up developing the study in a direction they don’t necessarily want it to go (02). • I would imagine the other factors would be the many considerations that go into the scientific side of designing a trial. I can imagine someone feeling a little bit overwhelmed at adding patient perspectives or worrying that patient perspectives may be in some ways at odds with the goals of a trial (07).
Facilitators	Education on value of patient perspectives	• I think having some sort of educational opportunities to let people know about this type of research and how to interpret it and how that interpretation looks different from, you know, the more traditional studies we’re used to reading is important. … Having more comfort potentially including some aspect of how to interpret this research in their training programs at the resident and fellow level… (02).
	Access to vetted patient experience information in a standardized format that doesn’t require a substantial time commitment	• I think there’s a wide and broad number of things that might be helpful…primarily focused on resources that would enable the acquisition of patient-centered outcomes. I could also see how a database like you are proposing to develop would be useful as a way to…collate or collect those experiences (12).
	Fostering change among funding sources	• I think, you know, the biggest thing is if the big granting organizations start to require that as an aspect of research, that certainly, I think, is extra impetus to make people forced into looking at these things. And, I think there is some aspect of that that’s starting to happen (02). • There are plenty of grant agencies and funding agencies that have stakeholders at the table for funding decisions. I just think the more we can incorporate and normalize it that would help (07).

### Past Experiences Incorporating Patient/Caregiver Perspectives into Clinical Research

Although the majority of participants were aware of the practice of integrating patient/caregiver perspectives into clinical research, past experience was quite varied, with some researchers reporting no experience and a similar number reporting extensive experience. Participants who had not used this practice overwhelmingly believed that implementing patient perspectives into their research would be beneficial.

### Barriers to Engaging Patients/Caregivers in Research

Participants reflected on a growing recognition among clinical researchers regarding the importance of understanding what matters most to patients and incorporating these aspects into their research. Yet, they also described existing barriers to doing so, including lack of knowledge or training in methods for patient/caregiver engagement, and the availability of time and resources to conduct patient/caregiver engagement in pediatric oncology research.

### Lack of Expertise on Methods for Patient/Caregiver Engagement

Many participants expressed that while physicians are trained in quantitative research methods, patient/caregiver engagement and research on patient/caregiver perspectives often fall into the realm of qualitative research, with few having had any training or knowledge in these areas. Participant 01 described it this way: “I’m building this solid tumor program and trying to really learn what our patients like about our program and what they don’t like…I find it hard to put together everyone’s opinions to really understand how to make something from these multiple opinions.” Further, many participants commented that while there is a growing awareness of research focusing on patient perspectives, there remains a general lack of awareness of how best to access and incorporate this information in a meaningful way.

### Availability of Time and Resources for Patient Engagement in Pediatric Oncology Research

Even if pediatric oncologists had the knowledge of how to obtain and utilize patient/caregiver perspectives for clinical research, several participants agreed that the availability of time and resources was a barrier. In addition, the primary goal of pediatric oncology clinical research is to improve survival rates for patients. While understandable, some participants thought this focus could hinder the inclusion of patient/caregiver experiences into clinical research design. Participant 09 summed it up by stating: “In some cases, they just don’t think the data gleaned from that type of investigation is worthwhile. Because they’re more centered on clinical outcomes like (EFS) [event free survival] and (OS) [overall survival]. The patient experience doesn’t matter. I’ve had this said to me explicitly. The patient experience doesn’t matter.”

Several participants noted that even for researchers who wish to incorporate patient perspectives into their research aims, this process can be overwhelming and may not always fit the research agenda nor priorities of the funding agency.

### Facilitators to Leveraging DIPEx as a Resource for Understanding Patient Perspectives and Experiences

Participants who had prior experience including patient perspectives in their clinical research believed it had been beneficial, as Participant 02 stated: “I’m on a research team looking at oto-protection and ototoxicity in patients being treated for cancer. We are just completing essentially a quality of life patient perspective study – that information is informing how we’re developing a study in the children’s group for medulloblastoma.” Yet, without qualitative expertise, such projects are challenging for most clinical researchers.

Identified facilitators that participants believe would promote the use of patient perspectives in clinical research included educational opportunities to better understand the qualitative methodology and how to assess the strength of evidence, as well as increased access to validated resources (e.g. methodologically rigorous sources of patient narrative data). Moreover, a cultural shift toward valuing and utilizing patient perspectives in a more uniform manner to better understand what matters most to patients has been gaining traction in the field of health research, including clinical research. Participants reflected on this growing trend and some suggested that if funding agencies requested such information as part of their grant proposal requirements, or if they prioritized clinical research that incorporated patient-centered outcomes, the uptake of including patient perspectives in clinical research would increase.

### Education on Value of Patient Perspectives

Multiple participants expressed an interest in having increased opportunities to learn more about the potential benefits of qualitative research and its methodology, patient-centered outcomes research, and methods for incorporating patient-centered research into their own research projects. Specific suggestions included increasing educational opportunities for evaluating qualitative research and dissemination strategies for promoting the importance of health experiences research in clinical research projects. Participant 07 suggested that “education and just a realization of the importance of doing this and seeing examples where it has been feasible to include patients and families in study design would go a long way.” Participant 08 emphasized a shift toward increasing patient/caregiver engagement in clinical research could be fostered by collaborations with pediatric oncology subfields that are already embracing this framework, such as palliative care, or with other departments such as social work.

### Access to Vetted Patient Experience Information in a Standardized Format That Doesn’t Require a Substantial Time Commitment

Overall, participants were enthusiastic about the prospect of utilizing DIPEx data as a resource for gaining insight into what matters most to patients/caregivers. Several participants felt that an increase in access to quality synthesized patient narrative data in a format that is easily accessible, such as a DIPEx web-based module, would aid their ability to incorporate patient perspectives into their research. Many participants also emphasized the value of leveraging a standardized and vetted approach. Participant 06 commented: “If there was a central way to get the information…where everybody could go from anywhere in the country and say this is what the data shows from the information parents and patients provided…would be ideal.” This approach could be leveraged to facilitate change in the types of questions that are included in clinical research, as Participant 05 stated: “Is there something beyond survival, are there complications that they [patients and their families] aren’t happy with, that they can’t live with, that had they known about, maybe they would have made other choices or done things differently?”

When asked about their preferred mechanism for accessing the data, some participants agreed that, while interesting, they may be reluctant to take the time to utilize the information available on a public-facing website. Instead, participants commented that a repository of indexed transcripts and/or peer-reviewed publications would be ideal. Several participants were candid about the reality that many clinical researchers would likely not have a significant amount of time to devote to mining a repository, however. For a DIPEx repository to be effective, data must be indexed in several different ways (e.g. experiences at different stages of treatment) to ensure easy access of information throughout the clinical research process.

## Discussion

To our knowledge, this is the first examination of interest in and feasibility of utilizing a repository of qualitative data on patient/caregiver experiences to inform clinical research in pediatric oncology. Because pediatric oncology clinical research is often focused on increasing survival, the opportunity to improve understanding of treatment side effects and their burden from patient/caregiver perspectives has the potential to enhance patient-centered care. Participants viewed the incorporation of patient experiences into clinical research as a key method for fostering patient-centered outcomes research. Participants described several factors that would support the use of patient/caregiver experiences in research, including access to quality synthesized patient narrative data in a centralized, easy-to-use format; educational opportunities to better understand qualitative research methodology; and the growing cultural shift among clinical researchers regarding the importance of understanding patient/caregiver perspectives and incorporating those aspects into their research. Our findings further highlight specific opportunities for increasing the utility of this resource. First, the discomfort described by clinical researchers in utilizing qualitative research and the lack of familiarity with assessing validity can be addressed by the development and dissemination of educational tools to assist individuals unfamiliar with qualitative methodology. Second, it is essential that data are organized in a manner that is user-friendly, enabling users to quickly access various types of information that could be implemented throughout a research project. A centralized repository of qualitative data on patient/caregiver experiences would streamline the process so that individual researchers would be able to forgo their own data collection, saving both time and resources, but this would need to be indexed appropriately to ensure ease of use. Finally, funding agencies have a critical role to play in supporting the development and use of high-quality qualitative data repositories, such as those produced using the DIPEx approach, to facilitate the integration of patient/caregiver perspectives into clinical research.

### Limitations

Our study has several limitations. Our sample is small, and although adequate to identify themes among a relatively homogenous group [20], larger studies examining perspectives across various clinical research disciplines would further illuminate facilitators and barriers to incorporating patient perspectives into clinical research processes. In addition, our recruitment methods may have introduced bias and it is possible that participants felt compelled to present favorable opinions on the potential of the DIPEx approach to inform the development of clinical research. Although participants could see the value of having a centralized resource that includes in-depth qualitative data from a broad range of patients/caregivers, user testing, and additional evaluation is needed to assess the utility of the DIPEx approach as a tool for informing the conception, design, and conduct of clinical research in practice.

## Supplementary material

For supplementary material accompanying this paper visit http://dx.doi.org/10.1017/cts.2021.822. click here to view supplementary material
