# Supplementary material for: Leveraging a qualitative data repository to integrate patient and caregiver perspectives into clinical research
Source: J Clin Transl Sci. 2021 Jul 21;5(1):e155. doi: 10.1017/cts.2021.822 (PMC8427548; doi:10.1017/cts.2021.822)
Supplement: Supplementary file 1 [file S2059866121008220sup001.docx]

**Views and Perceptions of the Utility of the DIPEx Method to Inform Pediatric Oncology Clinical Research**

1. Before we start, do you have any questions regarding DIPEx, health experiences research or the study that we are asking you to participate in?
2. Describe your current professional/academic position.
3. Have you ever been part of a team conducting a clinical trial or clinical research study? In what capacity?
   - 1. If yes, do you recall using information about patient and family perspectives to inform the conception, design or implementation of that research? How so?
4. In your opinion, what factors, if any, prevent oncology researchers from incorporating patient and family perspectives into their research?
5. What factors would facilitate oncology researchers to include patient and family perspectives into clinical research?
6. Are you familiar with research that focuses on health experiences from a patient or family’s perspective?
7. Could you envision using this type of resource (a library of patient narratives in or video and written transcript format) to help guide conception, design and implementation of clinical trials or clinical research studies?)
   1. If yes, when and how?
   2. If no, could you elaborate on why?
8. Are there any specific points in the research process in which you think this could be most useful?
9. Is there any specific information about patient experiences that you think would be helpful to gather or any specific questions that would be important for us to ask patients or their families?
10. What format would make information about patient perspectives or experiences most accessible and useful to you?
11. Do you have any ideas about how best to disseminate awareness of this resource to clinical researchers?
12. What other uses could you envision for this resource? (For example, educational tools; resources for patients/families; development of medical decision aids and medical guidelines; other uses in the clinical setting?)

1. Do you have any additional thoughts, advice or questions regarding DIPEx as a resource?
2. Do you have any recommendations for other pediatric oncology researchers we should interview?
